# Comparison of different concentrations of a povidone iodine-diluted sitz bath in the prevention of perianal infection in patients undergoing chemotherapy for hematological malignancy: study protocol for a randomized controlled trial

**DOI:** 10.1186/s13063-022-06721-y

**Published:** 2022-10-22

**Authors:** Yuqin Luo, Yingli Wang, Mei Yang, Ting Luo, Fengjiao Chen, Yamei Leng, Li Zhou, Jinbo Fang, Yuan Li, Chen Chen

**Affiliations:** 1 West China School of Nursing, Sichuan University/Department of Hematology, West China Hospital, Sichuan University, Chengdu, Sichuan China; 2 Department of Hematology, West China Hospital, Sichuan University, Chengdu, Sichuan China; 3 West China School of Nursing, Sichuan University, Chengdu, Sichuan China; 4 Cheng Du Shang Jin Nan Fu Hospital, West China Hospital, Sichuan University, Chengdu, Sichuan China

**Keywords:** Hematologic malignancy, Chemotherapy, Perianal infection, Povidone iodine, Sitz bath, Randomized controlled trial

## Abstract

**Background:**

Infection is one of the most common causes of death in patients with hematological malignancies during chemotherapy. Due to its special location, local warmth and humidity, repeated pollution with stool and urine, and characteristically wrinkled anatomical structure within which bacteria can hide, the perianal becomes a site with a high incidence of infection. Such infection also has a high recurrence rate and high mortality, increasing the economic burden of patients, delaying the time of treatment and reducing the quality of life. In severe cases, sepsis occurs and endangers the patient’s life. Previous studies have confirmed the effectiveness of povidone iodine (PI) in the prevention of perianal infection in patients with hematological malignancies during chemotherapy, but these reports have not documented in detail the adverse events associated with sitz bathing and the lack of randomized controlled trials of different concentrations of dilute povidone iodine sitz bathing. Therefore, the evidence is insufficient. Hence, the objective of this paper is to determine whether a povidone iodine diluent sitz bath can reduce the incidence of perianal infection compared with conventional perianal cleaning care and to observe the incidence of perianal infection, the severity of perianal infection, and the complications related to the sitz bath among groups treated with different concentrations of povidone iodine diluent, especially in high-risk patients prone to perianal infection, to screen for the optimal concentration.

**Methods:**

The trial is designed as a single-center, parallel, randomized, controlled and intervention trial with four parallel groups, and a primary endpoint of perianal infection occurred after this hospitalization chemotherapy. Randomization will be performed as simple randomization with a 1:1:1:1 allocation. This study received full ethics committee approval. The first patient was enrolled on May 1, 2021. A total of 268 patients with hematological malignancies undergoing chemotherapy who have risk factors for perianal infection will be enrolled with informed consent and randomly allocated to one of the four arms receiving (1) perianal cleaning care (control group D), (2) 1:100 PI diluted sitz bath (intervention group A), (3) 1:200 PI diluted sitz bath (intervention group B), and (4) 1:300 PI diluted sitz bath (intervention group C). The primary endpoint of the trial was the incidence of perianal infection. The secondary endpoints of the study will be the results of anal swab bacterial culture, the severity of perianal infection, the incidence of perianal adverse events (dryness, peeling, pigmentation, burning sensation), and pain scores. The length of hospitalization in days and hospitalization expenses will be recorded. Safety will be assessed with consideration of all adverse and severe adverse events related to the study treatment.

**Discussion:**

We hypothesized that patients with hematological malignancies during chemotherapy would benefit from a povidone iodine diluted sitz bath. This study will provide evidence-based recommendations for clinicians and nurses.

**Trial registration:**

Chinese Clinical Trial Registry (registration ID: ChiCTR2000041073). Registered on December 17, 2020. The protocol version number is V1.0,20201217. http://www.chictr.org.cn/edit.aspx?pid=66044&htm=4

**Supplementary Information:**

The online version contains supplementary material available at 10.1186/s13063-022-06721-y.

## Introduction

### Background and rationale

Hematological malignancies refer to diseases of abnormal proliferation of the hematopoietic or lymphocyte systems, accounting for nearly 10% of newly diagnosed cancers in the USA each year [1]. GLOBOCAN2020 statistics show that the incidence and mortality rate of hematological tumors are among the top 10 malignant tumors in the world [2]. Chemotherapy is still the main treatment at present. Patients with hematological malignancies are prone to infection complications, whether they have hematopoiesis disorders and immune function defects or neutropenia for a longer time after chemotherapy, among which perianal infection is common [3]. Bone marrow suppression is more common in patients with hematological malignancies after chemotherapy than in patients with solid tumors, and bone marrow suppression is an important factor in the occurrence of perianal infection, so the incidence of perianal infection caused by chemotherapy in patients with blood cancer is higher than that in patients with other cancers [4]. Previous studies have shown that the incidence of perianal infection can reach more than 40% in patients with hematological malignancies undergoing chemotherapy [5]. With the development of medical technology and management methods, the incidence has fluctuated from 6.7 to 27%, and the recurrence rate is as high as 31% [6, 7]; furthermore, perianal infection is one of the leading causes of death in patients with hematological malignancies, especially in patients with a lack of neutrophils. The mortality rate is between 11 and 57% [8, 9]. Perianal infection may prolong hospital stays, increase treatment costs, affect prognosis, and delay the timing of treatment; moreover, perianal pain, swelling, and other discomfort have a significant impact on patients’ quality of life and will endanger patients’ lives if sepsis occurs [10]. The treatment and nursing methods reported in previous studies mainly include Chinese herbal fumigation and sitting baths, wiping with acidified water, local antibiotic washing, wet compresses, microwave irradiation, and the use of new dressings [11–14]. Complex operation, difficulty obtaining materials, high economic cost, low compliance, increased workload of medical staff, and safety still need to be improved and are common drawbacks in previous studies.

Povidone iodine (PI) is a broad-spectrum antibacterial agent that is good at killing gram-positive bacteria, gram-negative bacteria, fungi, and viruses [15]. It is characterized by low irritation to the tissue and is suitable for skin and mucous membrane infections. Because of its strong bactericidal power, mild side effects, wide range of applications, and low price, it is favored by researchers. A previous randomized controlled trial included 124 patients with hematological malignancies undergoing chemotherapy. The results showed that a PI diluent sitz bath can effectively reduce the incidence and severity of perianal infection [16]. However, the incidence of comorbidities associated with sitz bathing has not been documented, and randomized controlled trials of sitz bathing with different concentrations of PI diluent are lacking. The results are limited to some extent. Therefore, there is no consensus on whether it is necessary to use a PI diluent in a sitz bath for patients with hematological malignancies during chemotherapy and what is the optimal concentration selection, especially in high-risk patients prone to perianal infection. The results of this randomized clinical study will initially help answer these questions.

### Objectives

We hypothesize that 1:300 is expected to be the minimum effective dose, and 1:100 is considered the maximum tolerated dose beyond which no further beneficial effect occurs. The primary objective of this randomized controlled clinical trial was to evaluate the efficacy of a PI diluted sitz bath in preventing perianal infection during chemotherapy in patients with hematologic malignancies, to screen for the optimal concentration and to detect a significant dose–response relationship in the primary endpoint change. Based on the evaluation of the clinical efficacy of the PI diluent sitz bath, we aim to explore its mechanism of action. To assess safety, the incidence of adverse events (AEs) and severe adverse events (SAEs) between the four study groups will be compared to illustrate the potential risk of diluted PI sitz baths for patients undergoing chemotherapy for hematological malignancies and provide more clinical evidence for the clinical application of the PI diluent sitz bath to prevent perianal infection.

## Methods

### Trial design

**Fig. 1:**
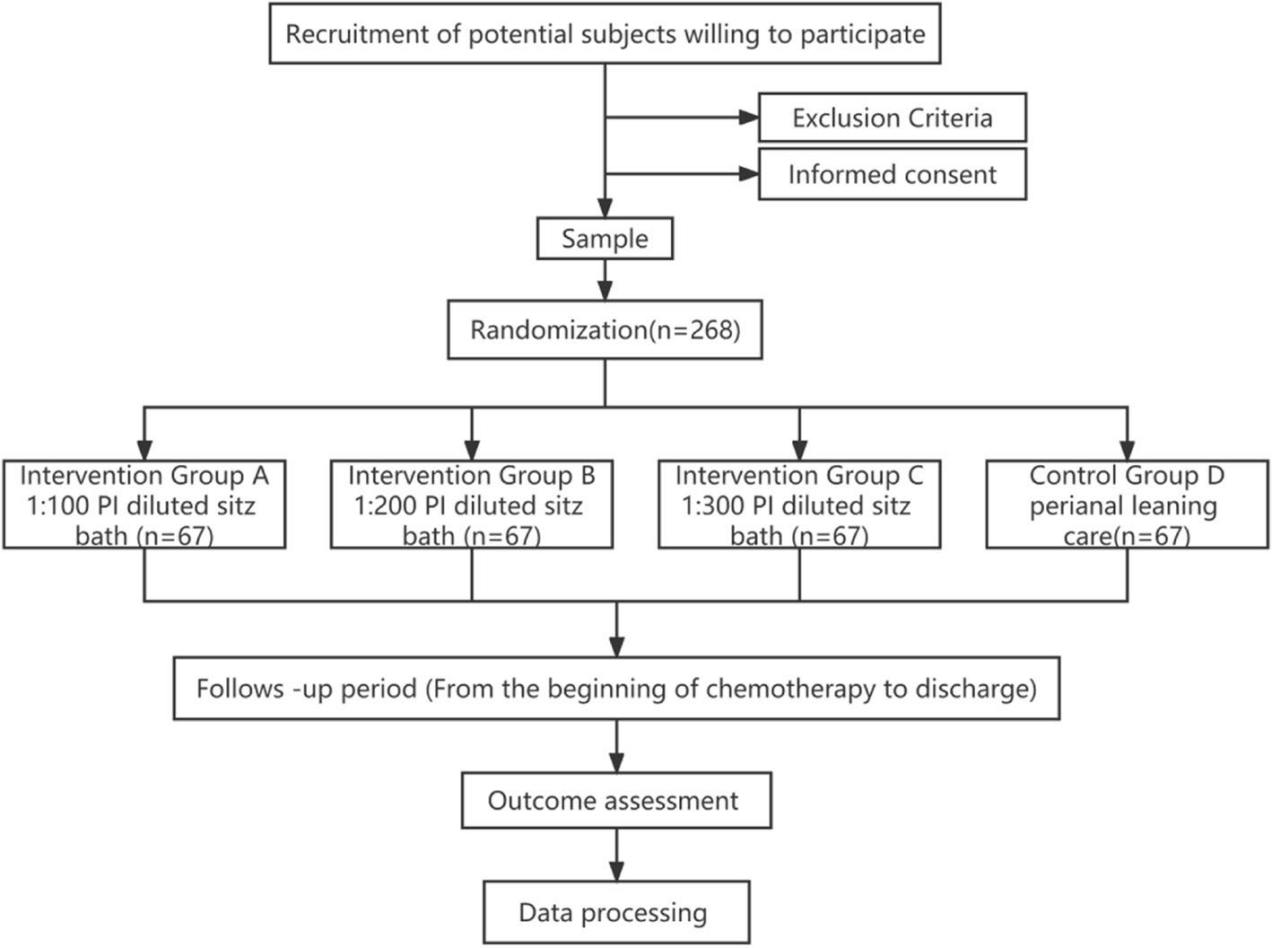
A flow chart of the study stages. PI, povidone iodine

**Fig. 2:**
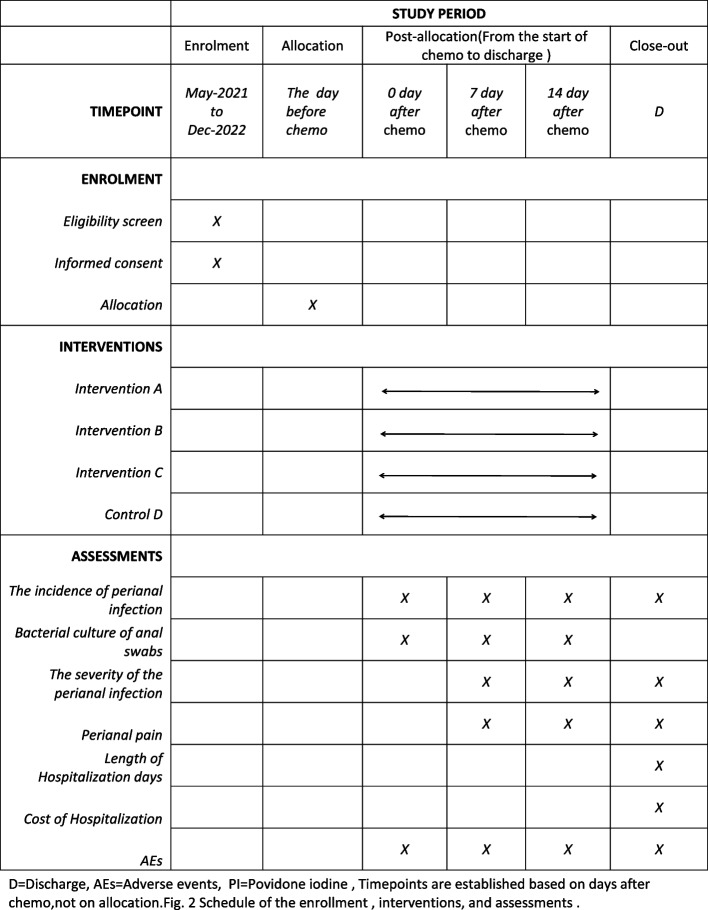
Timeline of the enrollment process, interventions, and assessments. D, discharge; AES, adverse events; PI, povidone iodine

This is a multiarm randomized controlled, single center, parallel, assessor-blinded superiority trial with a treatment allocation ratio of 1:1:1:1, which will be conducted in the Department of Hematology, West China Hospital, Sichuan University, China. Eligible participants who meet the diagnostic criteria for hematologic malignancy [17] will be enrolled (Fig. 1). The trial was approved by the Ethics Committee on Biomedical Research at West China Hospital of Sichuan University (approval code: NO. 2020-1170). It was also registered in the Chinese Clinical Trial Registry (registration ID: ChiCTR 2000041073), trial registration data in Supplement 1. A total of 268 patients with hematological malignancies undergoing chemotherapy will be randomly assigned to four groups. After randomization, patients in group D will receive conventional perianal cleaning care within 14 days after chemotherapy, while patients in the other three groups (A, B, and C) will receive a sitz bath consisting of a dilute PI solution at concentrations of 1:100, 1:200, and 1:300 on the basis of perianal cleaning within 14 days after chemotherapy. The timeline for the enrollment process, intervention, and follow-ups are summarized in a SPIRIT figure [18] (Fig. 2).

### Eligibility criteria {10}

#### Inclusion criteria

1. Meeting the diagnostic criteria of hematological malignancies;
2. Age of 18~80 years;
3. Hospitalized patients undergoing chemotherapy;
4. Having the willingness to cooperate during the study;
5. Having provided informed consent;
6. No perianal infection before chemotherapy;
7. Estimated hospital stay ≥ 14 days;
8. Patients with a history of perianal infection or patients with 3 or more of the following factors: age < 60 years, history of hemorrhoids, history of anal fissure, proneness to diarrhea, leukocyte promotion therapy before chemotherapy, or accepted a high dose of chemotherapy.
9. Note: Only the patients who met the above 8 items were included in the study.

#### Exclusion criteria

1. Chronic leukemia, Multiple myeloma, Crohn’s disease;
2. Allergy to iodine or PI;
3. Late pregnancy, postpartum two weeks, vaginal bleeding, pelvic acute infection;
4. Patients with mental disorders, an inability to cooperate with treatment, or other reasons for not completing the trial;
5. Patients in whom the curative effect cannot be determined or who have incomplete data.

### Sample size

This study has a randomized controlled design, and the evaluation index mainly involves the enumeration of data. The calculation formula of the sample size for multigroup rate comparison was adopted. In the sample size calculation, we used the chi-square test of the contingency table. The two-sided type I error probability alpha was set to 0.05, and the test power 1-beta was set to 0.9. The sample size ratio for each group was set to 1:1:1:1. In the preliminary trial, the incidence of perianal infection in patients with a history of perianal infection was 31.5%. The incidence rate of perianal infection was 26.7% in patients with three of the following five risk factors: age < 60 years, history of hemorrhoids, history of anal fissure, diarrhea within 1 week after chemotherapy, and white blood cell count less than 1 × 10^9^/L within 1 week after chemotherapy [19] (the relevant research results have been published). We set the incidence of perianal infection in the control group enrolled in this study was 25%. According to the results of the literature, the estimated incidence rate of perianal infection with a 1:100 dilution was 3.3%, which was set as the group with the best prevention effect. The rates of the other two groups were set as 20% and 10%. The overall effect size (W) of all groups was calculated to be 0.240 by the chi-square test. The chi-square distribution has 3 degrees of freedom. Taking into account the 10% loss to follow-up, the final sample size was 268 cases, with 67 participants per group.

### Study setting

This trial will take place at the Department of Hematology, West China Hospital, Sichuan University, China.

### Recruitment

Researchers will recruit participants from the inpatient wards of the Hematopathology Department. Patients eligible for the trial must meet all the inclusion criteria and exclusion criteria before enrollment. To recruit a sufficient number of participants, all doctors in these departments will be informed of the trial and will be asked to contact the research assistant if they encounter potentially eligible patients. Potential participants will be screened, will receive information on the trial, and then will be asked to sign informed consent forms. Patients who meet the selection criteria will undergo baseline assessments, and eligible patients will be asked by medical staff to complete a general information form including their name, sex, age, and medical history.

### Allocation

A simple random grouping method was used to number the patients according to the sequence of entering the experiment, to generate a random number for each subject (using the random number table in the Appendix of Practical Health Statistics of Peking University) and to sort the random numbers from small to large. The first 25% was set as group A, 25–50% as group B, 50–75% as group C, and the last 25% as group D. The corresponding experimental group or control group was entered according to the random number corresponding to the patient number. Group D was treated with traditional perianal cleaning care, and groups A, B, and C were treated with 1:100, 1:200, and 1:300 PI diluent sitting baths, respectively, on the basis of perianal cleaning care.

The random numbers generated were placed into sequentially coded, opaque, sealed envelopes. When the researchers determined that the subjects met the inclusion criteria, envelopes with the same number were opened according to the patient numbers so that the subjects could be assigned to the corresponding experimental groups.

The allocation sequencer was generated by the statistical analyst (not hematology staff), all members of the investigator team could register potential participants, and finally, the study leader individually assigned specific interventions to participants.

### Blinding

The third-party method of blindness was adopted, that is, the personnel who evaluated perianal conditions and the statisticians were blinded. The researchers and patients did not disclose any grouping information to the final efficacy evaluators and statistical analysis. If the participant had severe perianal infection or serious adverse events, unblinding was permissible. The evaluator was informed of the patient’s enrollment by the study leader, but the statistical analyst was not required to be informed.

### Relevant concomitant care permitted or prohibited during the trial

Four groups of patients will receive conventional treatment and care during the perioperative period. These treatments will include immune-boosting drugs before chemotherapy and anti-infective, leukocyte-boosting, pain-killing drugs, cooling, blood collection, and transfusions after chemotherapy, if necessary. All previous and concomitant medications will be administered as per clinical routine and according to the patients’ needs and demands. All anti-infectives, white blood cell boosters, and pain medications used will be recorded. The results will be recorded before and 2 weeks after chemotherapy and will include the protocell ratio, albumin level, white blood cell count, hemoglobin count, platelet count, blood culture results, and levels of C-reactive protein, procalcitonin, and interleukin-6. All team members will receive standard training on the test content, test design, test process and quality control before the start of the test. Interventions will be carried out in accordance with good clinical practice guidelines.

### Intervention description

#### Control group

In the control group, from the day of chemotherapy, the patients, when rising in the morning, before going to bed, and after defecation, will use warm water to clean the perianal area and will keep the perianal area clean and dry for 14 days. The cleaning method was to immerse the anus completely in warm water, gently wipe the anal skin with a towel, and then dry the anal skin. The water temperature will be 40–45°C, and the cleaning time will be 1–2 mi. The basic measures to prevent perianal infection are cleaning the anus and keeping it dry, which is also conventional perianal support treatment, so we chose this group as the control group.

#### Intervention group

Different concentrations of PI diluent (relative to the control treatment) will be added to the sitz bath. Patients will be randomly assigned to sitz baths with accurate PI concentrations. The duration of sitting bath treatment will be 14 days, the frequency of the sitting bath will be twice a day, the time for each sitting bath will be 10 min, the temperature of the sitting bath will be 40–45 °C, the height of the sit-down bath will be 40 cm, and the concentration of the sitting bath will be 1:100 (5% povidone iodine 20 ml + warm water 2000 ml), 1:200 (5% PI 10 ml+ warm water 2000 ml), or 1:300 (5% PI 6.7 ml+ warm water 2000 ml).

Most patients can recover from neutropenia in approximately 14 days. To facilitate the assessment and ensure the consistency of intervention, Zhou Ye, Lu Ling et al. [16, 20] conducted the perianal intervention for 14 days, which was the usual practice in previous studies, so the duration of perianal intervention was set as 14 days in this study . However, in some patients with acute myeloid leukemia, the duration of neutropenia and hospitalization is more than 14 days and up to approximately 28 days. For the special case, we will evaluate the risk factors for perianal infection after 14 days. We suggest that high-risk patients can continue to take the sitz bath, but there are no requirements on the concentration, duration and frequency of the sitz bath. At the same time, patients with hematologic malignancies are treated with systemic anti-infective therapy during myelosuppression.

#### Interventions: modifications

If participants are unable or unwilling to complete treatment or if a physician’s assessment indicates that other medical care interventions are necessary because of serious AEs or changes in their condition, further study interventions would not be appropriate. We would consider these participants to have given up treatment. The intervention can be modified at the request of the patient or at the recommendation of the doctor.

#### Interventions: adherence

Measures to improve patient compliance are as follows: first, health education should be carried out for patients to inform them of the benefits or necessity of perianal cleaning and sitz bath and the possible harm of not sitz bath to answer their questions and make patients fully informed, which is conducive to the guarantee of compliance; second, a perianal sitz bath inspector table should be set up so that patients or their family members can record the sitz bath situation every day in detail, including the specific time and water temperature; third, participants will be randomly checked daily for perianal sitz bathing and rewarded with incentives (e.g., masks) for high compliance.

#### Ancillary and post-trial care

All participants will return to usual care 14 days after the start of chemotherapy. As part of our safety protocol, we will monitor the patient for perianal skin allergies, dryness, peeling, and accidental injuries such as syncope and falls during the sitz bath. Any participants with the abovementioned abnormal situations will be contacted by the site coordinator, who will inform the doctor and researcher for timely treatment.

### Outcomes

#### Primary outcome

The primary outcome of interest will be the incidence of perianal infection during this hospitalization. Perianal conditions were examined by two researchers on day 0, day 7, and day 14 of chemotherapy and before discharge. Perianal infection occurred 14 days after chemotherapy and was immediately examined and recorded by the two researchers. The patient complained of perianal discomfort or was evaluated four times. If perianal infection occurs at any one time, it is considered that the patient has perianal infection. If the patient had no infection in each evaluation before discharge, it was considered that the patient had no perianal infection.

#### Secondary outcomes

The other outcomes of interest will be bacterial culture of anal swabs, the severity of the infection, perianal AEs, perianal pain, the length of hospitalization, and hospitalization expenses during this hospitalization. Bacterial culture of anal swabs will be performed on day 0, day 7, and day 14 of chemotherapy. The results of each bacterial culture will be recorded in detail, and descriptive analysis will be performed at days 0, 7, and 14 of chemotherapy. If a perianal infection occurs, two researchers will confirm the severity of the infection, with the final outcome measure being the most severe before discharge. Standard judgment of the degree of perianal infection is as follows [21]: degree i, redness, swelling, heat and pain; degree ii, obvious redness, swelling, heat and pain, abscess formation, sensation fluctuation at the top of the lesion; and grade iii, skin ulceration, necrosis, infection, bleeding, sinus or wound formation. Patients reporting perianal burning, peeling, dryness, and pigmentation will be recorded. If the patients do not report to the investigator, the incidence of perianal AEs in the patients will be observed on day 0, day 7, and day 14 of chemotherapy. Perianal pain will be measured at rest at 0, 24, and 48 h by using a visual analog scale (VAS) after perianal infection. The scale will range from 0–10 points, with higher scores indicating more severe pain [22]: 0 = no pain, 1~3 = slight pain that can be tolerated, 4~6 = pain that can be tolerated but affects sleep, and 7~10 = unbearable pain that affects not only appetite but also sleep. The length of hospitalization will be recorded in days. Hospitalization expenses will be recorded in yuan.

### Study organization

#### Data collection and management

The study will collect demographic and baseline functional information from the patients or the patient’s legally authorized representative. The data collection form can be found in the article written by Luo Yuqin et al. [19]. When the investigator contacted the patients or representatives, the unified guidance language was used to clearly inform the investigator’s identity and research purpose. The questionnaire part in this study was limited; the researchers collected information, asked for details of the patient and family members after completing all the entries, and confirmed them on site. Perianal infection, the primary endpoint, required a two-person assessment and signature confirmation, with a third person checking the electronic medical record for consistency. By referring to the data collected by the electronic medical record system, two uniformly trained hematology nurses will collect the information after the patient completes the sitz bath. One person will collect the data, and the other person will verify the data item by item to ensure that the data collection is correct. After verification, two people will input the Excel form. Finally, another researcher compared the data randomly checked with the electronic medical record system and paper files to achieve data consistency among the three parties.

The organizational structure of the trial is as follows. The steering committee will have full oversight over the design of the trial. The investigators of the Data Management Safety Committee (DMSC) will supervise and confirm that the case report form (CRF) is correctly completed and that the data are consistent with the original data. If there were any errors or omissions, the investigator immediately corrected them. For confidentiality, the electronic health information will be encrypted in accordance with the hospital protocol [23]. After the trial, personally identifiable information will be omitted and placed in a separate database for data analysis.

#### Statistical analysis

The validity analysis of the study will be mainly based on intention-to-treat (ITT, all randomized cases) and per-protocol analyses (PP, cases that comply with the trial protocol, good compliance, and completed CRF). First, the Shapiro–Wilk test will be used to test whether the quantitative data follow a normal distribution. The counting data are represented by examples and composition ratios. The measurement data conforming to a normal or nearly normal distribution are presented as the mean ± standard deviation, and the counting data not conforming to a normal distribution are presented as the median and quartile spacing. For the overall comparison of the rates of all groups, the chi-square test of the contingency table was used, and the Bonferroni method was used to adjust the probability of type 1 error for pairwise comparisons. The incidence of perianal infection and perianal AEs will be calculated by the chi-square test. If there were differences between the populations, the chi-square test was used, and the Bonferroni method was used to correct the differences between the groups. The Kruskal–Wallis rank-sum test will be used for the severity of perianal infection in general, and the Wilcoxon rank-sum test will be used for pial comparisons between groups if there are differences in population. Then, the relative risk reduction rate (RRR), absolute risk reduction rate (ARR), and number of patients requiring treatment (NNT) were calculated. Data will not be collected and analyzed for patients not undergoing chemotherapy. If scheduled chemotherapy is not performed, the study participant will be excluded from the clinical trial. This research will continue until the required number of evaluable patients is reached. Due to complications, a very small number of patients may not be able to comply with the randomly assigned treatment. The study will not exclude these patients. All statistical tests will be two-sided, and *P* < 0.05 will be considered statistically significant. In the safety analysis, Fisher’s exact test will be used to compare the incidence of AEs and SAEs by category (severity) between the two groups. The statistical analysis plan in Supplement 2.

If baseline factors were inconsistent between groups after randomization, confounding factors were controlled for by regression analysis. All data in this study were collected during the hospitalization of patients, with few missing data. If any data were missing, the multiple imputation method was adopted. The primary endpoint was evaluated using LOCF (last observation carried forward) analysis.

This study was a short-term clinical trial, and observation of the primary endpoint was completed during the hospitalization period. Conclusions could be drawn within a short period of time, unlike chemotherapy regimen selection, which had a long follow-up period. Therefore, there is no need for interim analysis and stop planning.

#### Adverse events (AEs)

In this study, AEs were defined as any treatment-related medical events, including any treatment-related adverse and unexpected signs, symptoms, or diseases. The AEs known to follow a sitting bath with a PI diluent solution include perianal skin pigmentation, burning sensation, dry skin, and peeling. At each visit, the participants will report AEs and be examined by a physician. AEs will be evaluated and reported to the principal investigator according to Common Terminology Criteria for Adverse Events (v4.03) [24].

#### Auditing

The ethics committee will conduct local monitoring of trial quality after the first patients have been enrolled. The trial management team will meet every 2 months to check the implementation of the study, including the recruitment rate, data quality, and adverse event reporting.

#### Plans for communicating important protocol amendments to relevant parties

The protocol, statistical analysis plan, data safety management plan, informed consent forms, and recruitment materials were reviewed and approved by the Ethics Committee on Biomedical Research at West China Hospital of Sichuan University. Any subsequent modifications will be submitted for review, and annual safety and progress reports will be presented. In addition, online trial registries will be updated accordingly.

#### Data access and dissemination plans

All principal investigators will be given access to the cleaned data sets. The data will be published on the website of the Clinical Research Management Department of West China Hospital of Sichuan University, and all data sets will be password protected. The data will be accessible through the research center upon reasonable request. The outcomes will be disseminated through peer-reviewed publications, a master’s thesis, community groups, or conference presentations. The PI should be considered for the lead author of this study.

## Discussion

Due to the special location of the perianal area, the local area is warm and wet and repeatedly polluted by urine and feces. The anatomical structural characteristics of perineal folds create conditions under which bacteria can hide, and the area can become a high incidence site of infection in patients with hematological malignancies undergoing chemotherapy.

Studies have shown that PI diluent sitz baths can effectively reduce the incidence of perianal infection; however, the complications associated with sitz baths have not been documented in detail, and randomized controlled trials of PI diluent sitz baths with different concentrations are lacking. This study will determine whether PI diluents should be routinely used in sitz baths for patients undergoing chemotherapy for hematological malignancies, especially in patients with high-risk factors for perianal infection, and will screen for optimal concentrations.

The whole process is simple, safe, comfortable and inexpensive. The test requires only the use of a measuring cup to measure warm water and the PI solution, followed by taking a sitz bath for 10 min and drying the perianal skin.

Methods to minimize bias are being implemented whenever possible. Randomization will be performed after admission and before chemotherapy. Randomization ensures a statistically consistent baseline between the experimental and control groups. The random number table in the Appendix of Practical Health Statistics of Peking University was used to generate random numbers, and patients were grouped according to the order of admission. Blinding patients is an important way to prevent bias; however, one limitation of this study is that the researchers and patients cannot be blinded to the group assignments. However, the observers and statisticians were blinded. Before participating in the study, the investigator must obtain and sign written informed consent from each participant.

In this study, improving patient compliance is a worthy issue. We have established a perianal sitting bath time form and a perianal sitting bath supervision form and will ask patients to register the daily sitting bath time and water temperature. Two research groups will regularly inspect the perianal conditions of patients to supervise them in following the prescribed concentration and frequency of the sitting baths. The research leader will bear full legal responsibility for the entire study and will publish the data on the website of the Clinical Research Management Department of West China Hospital of Sichuan University. Anyone who needs these data can contact the author. The email of the research leader is 409201462@qq.com.

## Trial status

Participant recruitment is still being undertaken. Enrollment started in May 2021, and the trial is expected to be completed by December 2022.This article is based on the protocol V1.0; 17.12.2020.

## Supplementary Information

**Additional file 1.** Trial registration data.

**Additional file 2.** SAP.

**Additional file 3.** Informed consent materials.

## Abbreviations

PI: Povidone iodine
AE: Adverse event
CI: Confidence interval
CRF: Case report form
ITT: Intention to treat
VAS: Visual analog scale
NRS: Numerical rating scale
D: Discharge
PP: Per protocol
RCT: Randomized controlled trial
SAE: Severe adverse event
SD: Standard deviation
LOCF: Last observation carried forward
