# Supplementary material for: Comparison of different concentrations of a povidone iodine-diluted sitz bath in the prevention of perianal infection in patients undergoing chemotherapy for hematological malignancy: study protocol for a randomized controlled trial
Source: Trials. 2022 Oct 22;23:895. doi: 10.1186/s13063-022-06721-y (PMC9587612; doi:10.1186/s13063-022-06721-y)
Supplement: Supplementary file 1 — Additional file 1. Trial registration data. [file 13063_2022_6721_MOESM1_ESM.pdf]

| **Trial registration data** | |
|---|---|
| **Data category** | **Information** |
| Primary registiyand trial identifying number | Chinese Clinical Trial Registry （ChiCTR2000041073 ） |
| Date of registration in primary registry | 17 Dec, 2020 |
| Registration Status： | 1008001 Prospective registration |
| Source of monetary or material support | West China Nursing Discipline Development Special Fund Project, Sichuan University |
| Primary sponsor | West China Hospital of Sichuan University |
| Secondary sponsors | West China Hospital of Sichuan University |
| Contact for public queries | Luo Yuqin,MSc Nursing,409201462@qq.com |
| Contact for scientific queries | Luo Yuqin,MSc Nursing,37 Guoxue Lane, Wuhou District, Chengdu, Sichuan, China |
| Public title | Study on prevention of Perianal infection in patients with hematologic malignancies by sitting bath with different concentrations of povidone iodine diluent |
| Scientific title | Study on prevention of Perianal infection in patients with hematologic malignancies by sitting bath with different concentrations of povidone iodine diluent |
| Countries of recruitment | China |
| Name of the ethic committee | Ethics Committee on Biomedical Research, West China Hospital of Sichuan University |
| Intervention(s) | Test group: 1:100 、 1:200 、1： 300 povidone iodine diluent sitz baths, respectively, on the basis of perianal cleaning care<br>Control group:perianal cleaning care |
| Key inclusion and exdusion criteria | Inclusion criteria: Meet the diagnostic criteria for hematologic malignancies;≧18 years and ≤ 80 years ; without perianal infection before admission; Patients admitted for chemotherapy; The expected length of hospital stay >= 14 days; Those who sign the informed consent. |

| | |
|---|---|
| | Exclusion criteria：Allergic to iodine or povidone iodine; Late pregnancy, two weeks after delivery, vaginal bleeding, pelvic acute infection; Patients with serious cardiac and renal diseases; Patients with mental disorders who cannot cooperate with treatment. |
| Study type | Interventional<br>Randomized, Intervention model, parallel assignment, Masking, single blind, Single center<br>Primary purpose: prevention<br>Phase Ⅳ |
| Date of first enrolment | May-2021 |
| Target sample size | 268 |
| Recruitment status | Recruiting |
| Primary outcome(s) | Incidence of perianal infection |
| Key secondary outcomes | Results of bacterial culture from perianal swabs;The severity of the perianal infection;The incidence of perianal adverse events (burning, peeling, pigmentation). |
