# Supplementary material for: Comparison of different concentrations of a povidone iodine-diluted sitz bath in the prevention of perianal infection in patients undergoing chemotherapy for hematological malignancy: study protocol for a randomized controlled trial
Source: Trials. 2022 Oct 22;23:895. doi: 10.1186/s13063-022-06721-y (PMC9587612; doi:10.1186/s13063-022-06721-y)
Supplement: Supplementary file 2 — Additional file 2. SAP. [file 13063_2022_6721_MOESM2_ESM.pdf]

SAP

(1) Sample size

1. Calculation method

The calculation formula of the sample size for multigroup rate comparison was adopted. In the sample size calculation, we used the chi-square test of the contingency table. The two-sided type I error probability alpha was set to 0.05, and the test power 1-beta was set to 0.9. The sample size ratio for each group was set to 1:1:1:1. In the preliminary trial, the incidence of perianal infection in patients with a history of perianal infection was 31.5%. The incidence rate of perianal infection was 26.7% in patients with three of the following five risk factors: age <60 years, history of hemorrhoids, history of anal fissure, diarrhea within one week after chemotherapy, and white blood cell count less than 1×10^9/L within one week after chemotherapy. We set the incidence of perianal infection in the control group enrolled in this study was 25%. According to the results of the literature, the estimated incidence rate of perianal infection with a 1:100 dilution was 3.3%, which was set as the group with the best prevention effect. The rates of the other two groups were set as 20% and 10%. The overall effect size (W) of all groups was calculated to be 0.240 by the chi-square test. The chi-square distribution has 3 degrees of freedom. Taking into account the 10% loss to follow-up, the final sample size was 268 cases, with 67 participants per group.

2. Sample size distribution

This trial is planned to enroll 268 subjects at a 1:1:1:1 ratio, including 268 patients in the control group and 68 patients in each intervention group.

(2) Analysis of data sets

Full Analysis Set (FAS)

All cases enrolled, with baseline data were included whenever possible. The absence of the main evaluation indicators in the FAS set will be filled by the last observation value carry-forward method (LOCF). All efficacy analyses will be performed in the FAS set.

per-protocol population set (PPS)

Refers to the collection of subjects who met the inclusion criteria, did not meet the exclusion criteria, and completed the test according to the protocol, that is, the cases who met the test protocol, had good compliance, completed the CRF requirements, and completed all the evaluations.

Security Data Set (SS)

Subjects receiving the intervention with documented data on safety indicators can enter the SS. Missing security data values must not be carried forward.

(3) Criteria for removing subjects

Before the statistical analysis of the data, the main researcher,the sponsor and the statistical unit shall judge whether the individual cases are excluded. In any of the following circumstances, the principal investigator should determine the degree to

which the subject has completed the trial and the reasons for the withdrawal. Determine whether to exclude this subject and explain it.

(1) The inclusion of the subject violates the inclusion / exclusion criteria and should not enter the test;

(2) During the trial, the investigator considers that the subject has other factors that is unable to continue to participate in the trial, and actually stops that the subject has to continue to participate in the trial;

(3) Subjects who change the treatment regimen during the trial;

(4) During the test period, the subjects do not comply with the test plan and have poor compliance, if the test products are not used, or the effectiveness and safety evaluation data cannot be collected according to the requirements of the test protocol.

(4) Statistical methods

All the statistical analysis was done using R 4.0.3. The validity analysis of the study will be mainly based on intention-to-treat (ITT, all randomized cases) and per-protocol analyses (PP, cases that comply with the trial protocol, good compliance, and completed CRF).For the overall comparison of the rates of all groups, the chi-square test of the contingency table was used, and the Bonferroni method was used to adjust the probability of type 1 error for pairwise comparisons. All statistical tests will be two-sided, and $P < 0.05$ will be considered statistically significant.

Main efficacy evaluation

The primary efficacy measures of this trial were the incidence of perianal infection, after completion of 2 weeks of treatment and before discharge.Relative risk

degree (RR values) and 95% confidence interval of perianal infection incidence in each intervention group and the control group.

H_0: incidence of perianal infection in each intervention group / perianal infection in control group> 1;

H_{α}: incidence of perianal infection in each intervention group/ incidence of perianal infection in control group 1.

The incidence of TOVA ACS perianal infections was calculated in the four groups, and the ratio (RR values) and 95% confidence intervals of the ratio between the three intervention groups and the control group were calculated, respectively. If RR = 1, there is no association between intervention factors and perianal infection.The RR> 1 and the 95% confidence interval did not contain 1, indicating that the intervention factor is a risk factor for perianal infection (a positive correlation).The RR 1 and the 95% confidence interval did not include 1, indicating that the intervention factor is a protective factor for perianal infection (a negative correlation).

Secondary efficacy evaluation

The secondary efficacy indicators of this trial are:

1. After 2 weeks of treatment, the relative ratio of the positive rate of anal swab bacterial culture between each intervention group and the control group was calculated as the formula of: relative risk= positive rate of anal swab bacterial culture in each intervention group / positive rate of anal swab bacterial culture in the control group.

2. Severity of perianal infection in each group after 2 weeks of treatment . If there is no infectionIt is 0 degrees, and if the infection occurs, it is divided into three levels: 1 degree, 2 degrees and 3 degrees.

3. Determine the perianal pain scores at 0h, 24h and 48h after perianal infection.

4. Days of hospitalization (days) of each group.

5. Hospitalization costs (yuan for each group).

Statistical description and inference of the secondary indicators will select the applicable description indicators and hypothesis testing method based on the data characteristics. The comparison of four groups of general situation will use appropriate methods according to the type of indicators, measurement data will compare the homogeneity of variance or Wilcoxon rank sum test, count data using chi square test or exact probability method (if chi-square test is not applicable), rank data using Kruskal-Wallis rank sum test.

Safety evaluation

The adverse reactions during the test were counted, and the specific adverse reactions that occurred in each group were recorded. Pieces, and analyze the number of occurrence cases and incidence rate, for description statistics. In the safety analysis, Fisher ' s exact test will be used to compare the incidence of AEs and SAEs by category (severity) between the two groups.

General information and baseline characteristics

The general information (demographic information) and the baseline clinical information were described.Statistical demographic and baseline characteristics of all

subjects: measurement data were described by mean (standard deviation) or median (interquartile). Group comparisons of measurement data were performed using group ANOVA or Wilcoxon rank sum test. The description of the count data or grade data will be used with various types of examples (percentage).Count data are compared by chi-square test or exact probability method (if chi-square test is not applicable). Grade data were compared between groups using the Kruskal-Wallis rank-sum test.

Results of laboratory tests

Some partial indicators of the blood routine of the enrolled population at each treatment stage were described. Memeasurement data (Standard deviation) or median (interquartile) description. Group comparisons of measurement data were performed using group ANOVA or Wilcoxon rank sum test. The grade data will be described with the number of various examples (percentage). Grade data were compared between groups using the Kruskal-Wallis rank-sum test.

(5) Processing of missing values and outliers

Missing value handling

The clinical trial process should be strictly controlled, and in principle, there should be no missing value. If missing values for baseline information, fill by multiple interpolation.If the subjects are included in the trial normally, the efficacy evaluation index should not be missing in principle. Subjects with missing the main efficacy index due to shedding will not be included in the PPS set. If the missing values occur during the clinical trial, they will be processed according to the analysis data set; the missing values of the main efficacy indicators will be filled by LOCF.
