# Supplementary material for: Comparison of different concentrations of a povidone iodine-diluted sitz bath in the prevention of perianal infection in patients undergoing chemotherapy for hematological malignancy: study protocol for a randomized controlled trial
Source: Trials. 2022 Oct 22;23:895. doi: 10.1186/s13063-022-06721-y (PMC9587612; doi:10.1186/s13063-022-06721-y)
Supplement: Supplementary file 3 — Additional file 3. Informed consent materials. [file 13063_2022_6721_MOESM3_ESM.pdf]

版本号：HX-IRB-AF-05-V3.0　　　　版本日期：2020 年 6 月 19 日

不同浓度聚维酮碘稀释液坐浴预防和治疗血液恶性肿瘤病人化疗期肛周感染的研究知情同意书

尊敬的受试者

我们邀请您参加四川大学华西医院批准开展的“不同浓度聚维酮碘稀释液坐浴预防与治疗血液肿瘤肛周感染效果观察”课题研究。本研究将在四川大学华西医院开展，估计将有 300 名受试者自愿参加。本研究已经得到四川大学华西医院生物医学伦理委员会的审查和批准。

1. 为什么要开展本项研究？

血液肿瘤疾病的常用治疗方法有化学药物治疗、标靶治疗及免疫性治疗，其中化学药物治疗是最常见的治疗方式，但长期化疗会使机体免疫功能、骨髓造血功能受到抑制，导致患者出现出血、感染等并发症，其中肛周感染是常见的感染疾病，在恶性血液肿瘤化疗患者中发病率能达到 40%以上，且肛周感染也是恶性血液肿瘤患者主要的死亡原因之一。患者发生肛周感染后会出现疼痛、发热、乏力等临床症状，影响化疗效果的同时对患者身心健康造成极大影响。肛周感染发生后患者易出现烦躁、焦虑等不良情绪，且肛周感染会延长患者住院时间，对患者及其家属造成沉重的经济负担。肛周感染主要以全身抗感染为主，但全身用药难以使药业直达病灶，此时肛周局部预防与治疗是关键。

文献回顾发现，对于肛周感染局部预防与治疗的方法比较多，比如：中药熏蒸、微波照射、局部抗生素冲洗、酸化水擦拭、高锰酸钾稀释液坐浴、甲硝唑坐浴、盐水坐浴、聚维酮碘湿敷、局部药物外用等。这些治疗方法存在操作时间长、不易操作、原材料不易获取、成本高、并发症多等问题。有研究已证实了聚维酮碘稀释液坐浴对于预防与治疗血液肿瘤肛周感染的效果，但是未对不同浓度聚维酮碘稀释液对于预防与治疗血液肿瘤肛周感染的效果进行比较。

本研究的目的是观察不同浓度的聚维酮碘稀释液坐浴对于预防与治疗血液肿瘤肛周感染的效果，寻求一种经济优效、易于操作、病人依从性好的预防与治疗肛周感染的方法。

2. 如果参加研究，您需要做什么？

如果参加本研究，您需要接受进行为期 14 天的肛周清洁或者 14 天聚维酮碘稀释液肛周坐浴。肛周清洗方法为肛周完全进浸没于温热水中，使用毛巾轻轻擦拭肛周皮肤，再擦干肛周皮肤，清洗用水量为 2000ml，水温为 40-45℃，清洗时间 1-2 分钟。肛周坐浴为在肛周清洁的基础上增加每日两次，早晚各一次，坐浴温度为 40℃-45℃，浓度分别为 1：100（5%聚维酮碘 20ml+温水 2000ml）、1：200（5%聚维酮碘 10ml+温水 2000ml）、1：300（5%聚

维酮碘 6.7ml+温水 2000ml）。您可能会被随机分配到肛周清洁组或者三个不同的试验组别中。在研究过程中，您会接受两次（研究开始前及研究结束时）问卷调查，以及在坐浴前一天，研究的第 7 天、14 天由专门经过培训过的人员观察您的肛周情况并记录；并在研究的前一天、第 14 天肛周清洁后进行肛周拭子细菌培养。

3. 可供选择的诊疗方案有哪些？

若您不愿意参加本研究的诊疗方案，您还可以采取甲硝唑坐浴、盐水坐浴、高锰酸钾坐浴、中药熏蒸等。

4. 哪些人不宜参加研究？

如果您有严重的心肾功能疾病、妊娠后期、产后两周、患有急性盆腔炎症及有阴道出血等，则不宜参加本研究。

5. 参加研究有哪些风险？参加本项研究，您可能会出现肛周皮肤色素沉着、肛周局部刺激烧灼感或者局部脱皮。如果在坐浴的时候，您出现任何不适，或者病情发生任何变化，或任何意外情况，不管是否与研究相关，均应及时通知您的医生、责任护士或研究者，她/他将会对此做出判断并给予适当的处理。因为聚维酮碘对局部组织和粘膜刺激较小，并在使用片刻后可以自行消失，无需进行特殊处理；且本研究中使用浓度较低，对局部粘膜刺激会更小，在使用药物片刻后或者停止用药、清洗局部皮肤后局部不良反应会消失，色素沉着及脱皮会好转。

6. 参加研究有哪些可能的好处？

参加本项研究，您的病情有可能获得改善，本项研究还有助于确定哪种治疗方法可以更安全有效地治疗与您具有相似病情的其他病人。

7. 参加研究需要支付有关费用吗？

本研究将公平、合理地选择受试者，对受试者参加研究不会收取任何研究费用，且不涉及任何额外的检查、治疗等，不会增加您的医疗、护理费用。如果出现与研究相关的损伤时，将依据国家有关规定提供相应的治疗与补偿。

8. 个人信息是保密的吗？

您的研究资料将保存在四川大学华西医院，研究者、研究主管部门、伦理委员会可查阅您的医疗记录。任何有关本项研究结果的公开报告将不会披露您的个人身份。我们将在法律允许的范围内，尽一切努力保护您个人医疗资料的隐私和个人信息。

9. 我必须参加研究吗？

参加本项研究是完全自愿的，您可以拒绝参加研究，或在试验的任何阶段随时退出本研究而不会受到歧视和报复，其医疗待遇与权益不受影响。如果您决定退出本研究，请与您的医生联系，以便妥善诊疗疾病。

受试者声明：我已经阅读了上述有关本研究的介绍，我的研究人员已向我充分解释和说明了本研究的目的、操作过程以及参加本研究可能存在的风险和潜在的获益，并回答了我所有相关问题。自愿参加本研究。

我同意☐ 或拒绝☐ 除本研究以外的其他研究利用我的研究资料和生物标本。

受试者正楷姓名：________________________

受试者签名：____________________________ 日期：__ __ __ __ 年 __ __ 月 __ __ 日

受试者的联系电话：______________________ 手机号：____________________________

法定代理人正楷姓名：______________________ （如适用）

与受试者关系：____________________________

法定代理人签名：__________________________ 日期：__ __ __ __ 年 __ __ 月 __ __ 日

需法定代理人签署的原因：____

见证人正楷姓名：______________________ （如适用）

见证人签名：__________________________ 日期：__ __ __ __ 年 __ __ 月 __ __ 日

需见证人签署的原因：____

医生声明：我已对上述参加本研究的自愿者说明了该项研究的有关细节，并且为他/她提供一份签署过的知情同意书的原件。我确认已向受试者详细解释了本研究的情况，特别是参加本研究可能产生的风险与受益、免费与补偿、损害与赔偿、自愿与保密等伦理原则和要求。

医生签名：_______________________________ 日期：__ __ __ __ 年 __ __ 月 __ __ 日

医生的联系电话：__________________________

四川大学华西医院生物医学伦理委员会　　联系电话：028-85422654，028-85423237
